# Pulsed Electric Field-Assisted Valorisation of Rosehip Seed Meal and Baobab Pulp to Produce a Fermented Beverage

**DOI:** 10.3390/molecules31152727

**Published:** 2026-08-06

**Authors:** Dorian Grillon, Awongwe O’theron Jonase, Thokozani Lumbo, Lusani Norah Vhangani, Laurent Thevenet

**Affiliations:** 1 Genie Biologique, Universite Paris Est Creteil, 94010 Créteil Cedex, France; dorian.grillon@agroparistech.fr (D.G.);; 2 Department of Food Science & Technology, Faculty of Applied Sciences, Cape Peninsula University of Technology, P.O. Box 1906, Bellville 7535, South Africa

**Keywords:** rosehip seed meal, baobab, valorisation, pulsed electric field, antioxidant activity

## Abstract

The growing demand for sustainable functional beverages has stimulated interest in the valorisation of agro-industrial by-products. This study investigated the effect of Pulsed Electric Field (PEF) treatment on the physicochemical properties, phenolic content, and antioxidant activity of a rosehip seed meal (RSM) and baobab pulp (BP) fermented beverage. RSM was subjected to PEF of 1.5 and 3.0 kV/cm. A yeast (*Saccharomyces cerevisiae*)-fermented beverage was formulated with RSM, BP and other ingredients. Physicochemical parameters—including pH, total soluble solids (°TSS), alcohol by volume (%ABV), and colour (L*, a*, b*)—were evaluated together with total phenolic content (TPC), DPPH radical scavenging activity, and ferric reducing antioxidant power (FRAP). Fermentation significantly (*p* < 0.05) decreased TSS and increased %ABV content, confirming microbial metabolism, while pH remained relatively stable throughout the process. Colour changes involving an increased lightness and decreased red and yellow intensities were observed after fermentation. TPC increased significantly (*p* < 0.05) from 499.30 to 602.41 mg GAE/L, with the highest value observed for 3.0 kV/cm after 24 h fermentation. Similarly, FRAP values increased from 362.49 to 467.55 µmol TE/L, whereas DPPH activity remained constant. The results demonstrate that PEF pretreatment can enhance fermentation by increasing the extraction of phenolic compounds, thus affecting the antioxidant potential. This highlights the potential for PEF as a green technology for developing sustainable, value-added functional beverages.

## 1. Introduction

The increasing global demand for sustainable food systems has intensified interest in the valorisation of agro-industrial by-products as alternative raw materials for developing functional foods and beverages [1]. Large quantities of by-products generated from fruit-, vegetable-, cereal-, and food-processing industries are often underutilised or discarded, despite containing substantial amounts of biologically active compounds, including polyphenols, dietary fibre, vitamins, minerals, carotenoids, and organic acids [2]. The disposal of these materials not only represents a loss of valuable nutritional resources but also contributes to environmental challenges associated with waste management. Consequently, the conversion of agro-industrial by-products into value-added products has emerged as an important strategy for promoting sustainability, reducing food waste, and improving resource efficiency in the food sector [3].

The valorisation of agro-industrial waste aligns closely with the circular economy, where the value of the resources must be maintained within production systems for as long as possible while minimising waste generation [2,4]. Furthermore, such approaches contribute directly to several United Nations Sustainable Development Goals (SDGs), including SDG 2 (Zero Hunger), SDG 3 (Good Health and Well-being), SDG 9 (Industry, Innovation and Infrastructure), SDG 12 (Responsible Consumption and Production), and SDG 13 (Climate Action) [5]. Within the African context, the development of sustainable value-added products from locally available resources also supports the aspirations of the African Union Agenda 2063, particularly the goals of inclusive economic growth, industrialisation, sustainable natural resource utilisation, food security, and the advancement of science, technology, and innovation as drivers of socioeconomic development [6].

Among the various approaches for by-product valorisation, fermentation represents one of the oldest and most effective biotechnological processes for enhancing the nutritional, functional, sensory, and shelf-life characteristics of food products. Fermented beverages constitute an important segment of the global food market. They may be produced from cereals, fruits, vegetables, tea leaves, and other plant-derived substrates through the action of yeasts, bacteria, or mixed microbial cultures [7,8,9]. These beverages are commonly classified according to alcohol content as alcoholic > 1.2% alcohol by volume (ABV), low-alcoholic ≤ 1.2%, and non-alcoholic < 0.5% [10,11]. In recent years, growing consumer awareness of health, sustainability, and knowledge of dietary needs has stimulated the development of plant-based fermented beverages as alternatives to conventional dairy and alcoholic products. Such beverages offer opportunities to deliver bioactive compounds, antioxidants, vitamins, minerals, probiotics, and other health-promoting constituents while simultaneously utilising non-conventional raw materials and processing by-products.

Advances in food-processing technologies have further enhanced the potential of fermentation-based product development. Conventional thermal processing methods may negatively affect heat-sensitive compounds, resulting in losses of nutritional quality, flavour, colour, and biological activity [2]. Consequently, increasing attention has been paid to greener, more sustainable non-thermal technologies that improve process efficiency while preserving product quality. Among these technologies, Pulsed Electric Field (PEF) processing has emerged as a promising green technology for food and beverage applications. PEF is a non-thermal technology that applies short-duration high-voltage electrical pulses to biological materials, causing electroporation of cell membranes [2,12]. This phenomenon increases membrane permeability and facilitates the release of intracellular compounds without the detrimental thermal effects associated with conventional processing methods. As an emerging non-thermal technology, PEF offers superior extraction capabilities while reducing energy requirements and preserving thermolabile compounds. Previous studies have demonstrated that PEF enhances mass transfer processes, improving the extraction of phenolic compounds, pigments, volatile compounds, antioxidants, and other valuable intracellular constituents from plant materials [1]. Positive effects of PEF have been reported for various agricultural commodities, including apples, grapes, berries, and hop pellets, with improved extraction yields and enhanced functional properties observed. Furthermore, PEF has shown potential to improve fermentation performance by increasing the availability of fermentable substrates and nutrients for microbial metabolism, thereby promoting the production of desirable metabolites and shortening fermentation times [13].

Rosehip seed meal, a by-product of rosehip oil extraction, is an underutilised resource with considerable potential for valorisation. Although frequently discarded as waste, rosehip seed meal contains significant quantities of dietary fibre, polyphenols, antioxidants, vitamins, and essential fatty acids that may be exploited in the development of functional food products [14]. Similarly, baobab (*Adansonia digitata* L.) pulp is recognised as a nutrient-dense African superfruit rich in vitamin C, minerals, dietary fibre, and antioxidant compounds. The incorporation of baobab pulp into fermented beverages offers opportunities to enhance both nutritional quality and sensory appeal while promoting the utilisation of indigenous African resources [14,15,16].

The development of a fermented beverage incorporating rosehip seed meal and baobab pulp offers an innovative approach to valorising agro-industrial by-products while supporting sustainable food-production systems. By combining the bioactive potential of rosehip seed meal with the nutritional richness of baobab pulp and the process-enhancing capabilities of PEF technology, it is possible to create a novel functional beverage that aligns with contemporary consumer demands for health-promoting, environmentally sustainable, and minimally processed products. Such an approach not only contributes to waste reduction and resource efficiency but also demonstrates the practical application of circular economy principles within the food industry.

The application of PEF technology has been increasingly investigated to valorise agro-industrial by-products to produce functional fermented beverages. However, to the best of our knowledge, PEF valorisation of rosehip seed meal, particularly in combination with baobab powder, has not been studied. Therefore, this represents a research gap, as the potential of this underutilised by-product and baobab combination remains largely unexplored.

Therefore, this study aimed to investigate the effect of PEF treatment and fermentation time on the physicochemical properties, phenolic composition, antioxidant activity, and fermentation characteristics of a novel rosehip seed meal–baobab fermented beverage.

## 2. Materials and Methods

### 2.1. Chemicals

Rosehip seed meal and baobab pulp were supplied by Bioslyx PTY LTD (Paarl, Cape Town, South Arica). 2,2-Diphenyl-1-picrylhydrazyl, absolute ethanol, sodium hydroxide, hydrochloric acid, ascorbic acid, methanol, acetic acid, chloroform, potassium iodide, sodium thiosulfate, potassium hydrogen phosphate, absolute ethanol, ascorbic acid, potassium ferricyanide, trichloroacetic acid, potassium persulfate, sodium chloride, ferric chloride and hexane were purchased from Merck (Modderfontein, Johannesburg, South Africa). Folin and Ciocalteu’s phenol reagent, gallic acid monohydrate, and 2,4,6-tri [2-pyridyl]-s-triazine, ascorbic acid, quercetin and luteolin were purchased from Sigma-Aldrich (Kempton Park, Johannesburg, South Africa). Yeast (*Saccharomyces cerevisiae*), (Anchor Brewer’s Yeast, Inkunzi Malanga, Goodwood, Cape Town, South Africa) used as the fermentation agent in this study, was bought at a local supermarket Spar (Bellville, Cape Town, South Africa). All chemicals used in this study were analytical grade, and chemical reagents were prepared according to standard analytical procedures. Prepared reagents were stored under conditions that prevented deterioration or contamination. The water used in this study was ultrapure water purified with a Milli-Q water Millipore purification system (Microsep, Bellville, Cape Town, South Africa).

### 2.2. Pulsed Electric Field-Assisted Extraction of Rosehip Seed Meal

Pulsed electric field treatment was performed according to Boussetta et al. [17], with slight modifications. A floor-standing bench PEF system (DIL-Elea, Quakenbrück, Germany) was used to generate exponentially decaying pulses with a maximum voltage of 30 kV at a pulse frequency of 1 Hz. Defatted ground rosehip seed meal in water 1:9 mass/vol was placed in an 8 cm electrode gap treatment chamber exposed to an electric field strength of 1.5 and 3 kV/cm resulting in specific energies of 15 and 30 kJ/kg, respectively. The untreated seed meal (control) was stored in water simultaneously to ensure comparability. The PEF-treated and untreated seeds were freeze-dried (SP Scientific, Warminster, PA, USA) and stored in airtight bags until use.

### 2.3. Preparation of the Rosehip Baobab Fermented Beverage

Three RSM treatments were prepared: an untreated control, PEF treated at 1.5 kV/cm (15 kJ/kg), and PEF treated at 3 kV/cm (30 kJ/kg). A beverage formulation was prepared using water (80%), RSM (10%), baobab pulp (2%), sugar (7%) and yeast (1%). The RSM flour was mixed with boiled water, sugar, and pulp, then boiled for 10 min with continuous stirring. The resulting solution was cooled to 25–30 °C. A mixture of yeast (*Saccharomyces cerevisiae*) and sugar at a 1:1 ratio was dissolved in water (35–40 °C) and allowed to stand for several minutes. This preparation was based on the modified method of Gao et al. [8]; after which, the boiled rosehip and the yeast mixture were combined and stirred. Of the cooled beverages, one half was immediately filtered, gassed and rested at 5 °C. This was designated as the 0 h fermentation samples (T_0_). The second portion was transferred into a fermentation flask fitted with a CO_2_ trap (airlock) containing 70% (*v*/*v*) ethanol to permit the escape of carbon dioxide while minimising the risk of contamination. The mixture was allowed to ferment for 14 and 24 h at 30 °C in an incubator (Memmert, Schwabach, Germany). Following fermentation, the slurry was filtered using a simple laboratory-scale pressing method. The slurry was transferred into a sterilised food-grade muslin cloth, tied, and placed into a cheese mould (Kadova, Berkel en Rodenrijs, The Netherlands) fitted with a pressing lid suspended over a stainless-steel collection bowl. The mould lid was applied with manual pressure. Filtration was complete when no further liquid could be recovered. The filtered beverage was gassed and rested at 5 °C and designated as the 14 and 24 h fermentation samples (T_14_ and T_24_). The experimental design, therefore, consisted of three RSM treatments (control, 1.5 kV/cm, and 3 kV/cm) and three fermentation times (0, 14 and 24 h), resulting in a total of 9 treatment combinations.

#### 2.3.1. Physicochemical Properties of the Beverage

The pH of the beverage was measured using a calibrated 913 pH meter (Metrohm, Herisau, Switzerland). Total soluble solids (TSS%) as °Brix were measured with a refractometer. The colour of the beverage was measured according to the modified method of. The CIELab parameters L* (Lightness, 100 = white, 0 = black), a* (+red; −green) and b* (+yellow; −blue) were measured with a spectrophotometer (CM-5, Konika Minolta, Japan) using a D65 daylight source, a large viewing area and an observer angle of 10°. Measurements were performed in triplicate, and the system reported the average. The alcohol by volume (ABV%) was calculated using the following formula:

ABV% = (SG_before_ − SG_after_) × 131.25

where SG refers to the specific gravity measured before and after fermentation.

#### 2.3.2. Determination of Total Phenolic Content (TPC)

The Folin–Ciocalteu assay for the determination of TPC was adopted from the study conducted by Vhangani et al. [18], with slight modifications. The beverage samples were diluted with water at a 1:6 ratio and then centrifuged for 10 min at 3600 rpm. 0.5 mL of the resulting supernatant was mixed with 2.5 mL of Folin–Ciocalteu reagent (diluted tenfold) and 2 mL of sodium carbonate solution (7.5%). The absorbance of the mixture was measured at 765 nm after 30 min using a spectrophotometer (Lambda 25, PerkinElmer, Singapore). A calibration curve was drawn with gallic acid standards (5–200 μg·mL^−1^). The TPC was expressed as Gallic acid equivalent (mg GAE·g^−1^ of dry material).

#### 2.3.3. Determination of 1,1-diphenyl-2-picryl-hydrazyl Radical Scavenging (DPPH-RS) Activity

The DPPH radical-scavenging capacity was determined according to the method of Dhlakama et al. [19], with slight modifications. The beverage samples were diluted with water at a 1:6 ratio. Subsequently, they were centrifuged for 10 min at 3600 rpm. An 800 μL aliquot of the supernatant was mixed with 4 mL of 0.12 mM DPPH in absolute methanol solution. The mixture was vortexed and allowed to stand at room temperature in the dark for 30 min. The absorbance of each of these sample solutions was measured at 517 nm in a spectrophotometer (Lambda 25, Perkin Elmer, Singapore) using a 1.5 cm path-length glass cuvette. The control was prepared in the same manner, but the sample solution was substituted with water. The percentage of inhibition was calculated using the formula

% DPPH − RS = [(A_0_ − A_1_)/A_0_] × 100

where A_0_ is the absorbance of the control, and A_1_ is the absorbance of the beverage sample at 517 nm.

#### 2.3.4. Ferric Reducing Power Assay

The rosehip baobab ale samples were diluted with water at a 1:6 ratio. Subsequently, they were centrifuged for 10 min at 3600 rpm. FRAP was determined as described by Dhlakama et al. [19], with slight modifications. An amount of 0.5 mL of the supernatant was mixed with 1.25 mL of 0.2 M Phosphate buffer at pH 6.6 and 1.25 mL of 1% potassium ferricyanide. The reaction mixture was incubated in a water bath (Lasec, Ndabeni, Cape Town, South Africa) at 50 °C for 30 min, followed by the addition of 1.25 mL of 10% trichloroacetic acid. Then, 1.75 mL of Mill Q water and 0.85 mL of 0.1% ferric chloride were added. The blank was prepared by adding all the above-mentioned reagents, by substituting the sample with water. The absorbance of the reaction was measured at 700 nm using a spectrophotometer (Lambda 25, Perkin Elmer). Ascorbic acid was used as a reference antioxidant. The calibration curve was established using the following concentrations: 10, 25, 50, 75 and 100 µg/mL of Ascorbic acid solutions. The reducing power will be expressed as an increase in absorbance at 700 nm.

### 2.4. Statistical Analysis

Statistical analysis was performed using SPSS 29.0 for Windows^®^. Analysis of variance (ANOVA) established the significance of each dependent factor. Descriptive statistical analyses determined the triplicate’s mean and standard deviation (*n* = 3). Duncan’s multiple range post hoc test was used to indicate significant differences among the means. The level of confidence required for significance was selected at 95%.

## 3. Results and Discussion

### 3.1. Physicochemical Properties

#### 3.1.1. pH

**Figure 1:**
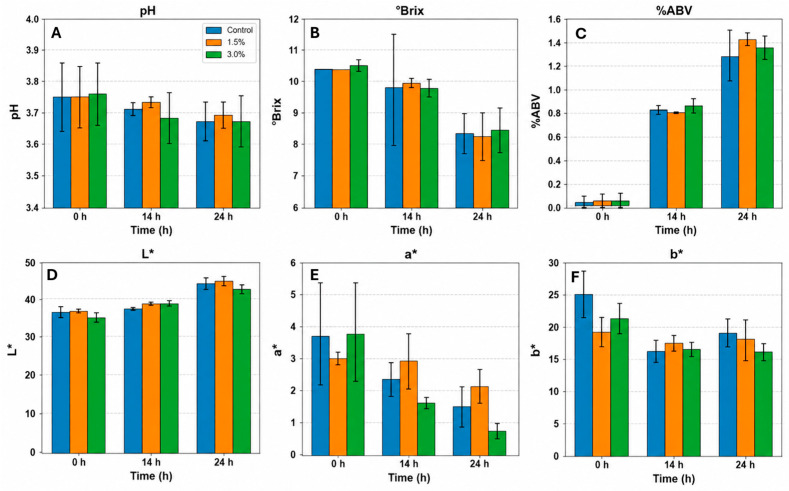
(**A**–**F**): Physicochemical properties of PEF-treated rosehip seed meal and baobab pulp fermented beverage. (**A**): The effect of PH. (**B**): Total soluble solids (Brix). (**C**): Alcohol by volume (ABV). (**D**): Lightness (L*). (**E**): Red/green. (**F**): Yellow/blue colour coordinates.

The pH values of beverages are depicted in Table 1 and Figure 1A. The pH values ranged from 3.67 to 3.76 and did not differ significantly among treatments or fermentation times (*p* > 0.05). The absence of significant changes indicates that PEF treatment at 1.5 and 3.0 kV did not substantially affect the beverage’s acidity. Although a slight decrease in pH was observed after fermentation, particularly at 24 h, the changes were minimal. This suggests that the production of organic acids during fermentation was insufficient to cause marked alterations in pH, likely due to the buffering capacity of constituents and the relatively short fermentation period. Our results contradict those of Sun et al. [20], who reported a reduction in pH from 4.55 to 4.08 during 48 h of fermentation of apple juice due to the accumulation of organic acids. Maintaining low pH values throughout fermentation is beneficial for microbial stability and product safety. Moreover, the relatively stable pH observed during fermentation may also be linked to the beverage’s initially acidic nature. Unlike many fruit-based fermentations that begin at higher pH values and undergo substantial acidification [8,20], our beverage already possessed an acidic environment favourable for fermentation and microbial stability. Consequently, only minor pH changes were observed despite evidence of active sugar utilisation and ethanol production.

#### 3.1.2. Total Soluble Solids (°Brix)

The °Brix values as depicted in Table 1 and Figure 1B decreased significantly from approximately 10.40–10.53 at 0 h to 8.23–8.43 at 24 h (*p* < 0.05). This reduction reflects the utilisation of fermentable sugars by microorganisms during fermentation, resulting in the conversion of sugars into ethanol and other metabolites [21]. No significant differences were observed between the control and PEF-treated samples at each fermentation time, indicating that PEF treatment did not significantly affect sugar consumption. The similar decline across all treatments suggests that microbial fermentation proceeded at comparable rates regardless of PEF exposure. Similar decreases in °Brix have been reported in yeast-fermented herbal beverages [22], fruit wines [23], and mead products [21], where sugars were converted into ethanol, carbon dioxide, and other fermentation metabolites. The reduction from 10.5 °Brix at 0 h to 8.3 °Brix after 24 h in the present study corresponds with the increase in alcohol content, confirming the progression of fermentation. These findings suggest that the fermentative microorganisms efficiently utilised available carbohydrates regardless of PEF treatment, resulting in comparable sugar consumption patterns among the treatments.

#### 3.1.3. Alcohol Content (%ABV)

Table 1 and Figure 1C depict the %ABV of fermented beverages. The alcohol content increased significantly with fermentation time, rising from negligible levels of 0.03–0.04 at 0 h to 0.80–0.85 at 14 h and 1.28–1.42% ABV at 24 h (*p* < 0.05). Based on the %ABV, our beverage falls under the low-alcohol-to-alcoholic fermented beverage category, 0.8–1.42% [1,24]. This increase corresponds to the metabolic conversion of sugars into ethanol by fermentative microorganisms. The PEF-treated samples exhibited alcohol concentrations similar to those of the control at each fermentation stage, indicating that PEF treatment did not significantly (*p* > 0.05) alter ethanol production. Nevertheless, the 1.5 kV treatment showed the highest alcohol content at 24 h (1.42%), suggesting that moderate PEF exposure may have slightly enhanced fermentation activity, although the differences were not statistically significant. The enhanced fermentation performance, as evidenced by reduced sugar content and increased alcohol content in PEF-treated samples, may be associated with increased nutrient availability resulting from electroporation-induced cell permeabilisation. Similar findings were reported for PEF-treated rosehips, where the release of intracellular constituents improved substrate accessibility and supported microbial activity during fermentation [1].

#### 3.1.4. Colour (L*, a*, b*)

The lightness (L*) values generally increased during fermentation, indicating that the beverages became lighter in appearance over time (Table 1 and Figure 1D). At 0 h, lightness (L* values) ranged from 34.70 to 36.55, while values increased to 41.33–43.68 after 24 h of fermentation. The increase in lightness may be attributed to the transformation of coloured compounds such as polyphenols during microbial activity. Among the treatments, the 3.0 kV/cm sample at 0 h exhibited the lowest L* value, suggesting a darker appearance immediately after PEF treatment [24]. However, after fermentation, all samples showed increased lightness in proportion to the initial values for each treatment, demonstrating that fermentation had a greater effect on colour development than the PEF treatment [25,26]. Contrary to our results, Tan et al. [25] reported no change in L* value of pumpkin juice after 27 h of fermentation, but a significant decrease was observed for the a* and b*. In the current study, the a* values decreased during fermentation, indicating a reduction in red colouration. Initial values ranged from 2.94 to 3.65 at 0 h but declined to 0.76 to 2.10 after 24 h (Table 1 and Figure 1E). Significant reduction (*p* < 0.05) in a* was only observed between 0 and 24 h for RSM_C and RSM_3.0, whilst no change was observed for RSM_1.5. (*p* > 0.05). Moreover, the a* value reduction was 59% and 79% for RSM_C and RSM_3.0, respectively, suggesting that higher PEF intensity combined with fermentation contributed to a greater reduction in red pigments. We speculate that the decline may result from enzymatic oxidation, polymerisation, or microbial transformation of phenolic compounds and pigments present in the beverage. The reduction in a* values corresponds with the observed increase in lightness and indicates progressive colour modification during fermentation. A testament to our results is the reduction in redness of fermented beverages such as pumpkin juice [25], bayberry [27], kombucha and pineapple juice-based kombucha [26], which the authors attributed to microbial metabolism, pigment transformation, and substrate utilisation. The b* values showed a decreasing trend due to fermentation, falling from 16.92–20.80 at 0 h to 13.65–15.87 at 24 h (Table 1 and Figure 1F). Similar to the results reported for lightness, the reduction (*p* < 0.05) in yellowness was only observed after 24 h of fermentation between RSM_C and RSM3.0. This reduction indicates a loss of yellow colouration over time; it may be associated with the hydrolysis of pigments and phenolic compounds during fermentation, as well as with potential oxidative reactions that affect colour stability. The lower b* values at 0 h fermentation observed in PEF-treated samples suggest that PEF may have facilitated structural changes in colour-related compounds. However, fermentation remained the primary factor influencing colour evolution.

### 3.2. Phenolic Content and Antioxidant Activity

#### 3.2.1. Total Phenolic Content (TPC)

The TPC of fermented beverages ranged from 499.30 to 602.41 mg GAE/L and showed a significant increase during fermentation (*p* < 0.05) (Table 2). At 0 h, the control sample exhibited the lowest TPC (499.30 mg GAE/L), while the 3.0 kV/cm PEF-treated sample showed a slightly higher value (520.00 mg GAE/L). Following 14 h of fermentation, TPC increased in all treatments, reaching values between 553.00 and 570.67 mg GAE/L. After 24 h, the highest TPC was observed in the 3.0 kV/cm treatment (602.41 mg GAE/L), which was significantly higher than the control at 0 h. The increase in TPC during fermentation may be attributed to microbial enzymatic activities that hydrolyse complex phenolic compounds and release bound phenolics from the plant matrix, thereby increasing their extractability and detection [8].

Furthermore, PEF treatment may have enhanced cell membrane permeabilisation and facilitated the release of intracellular phenolic compounds. The combination of PEF and fermentation has exerted a synergistic effect, particularly at 3.0 kV/cm and 24 h, resulting in the highest phenolic concentration among all treatments. Our results are similar to those reported by Ntourtoglou et al. [28] for PEF-treated rosehips; they reported that an increase in TPC may be attributed to electroporation-induced permeabilisation of plant tissues, which enhances the release of intracellular phenolic compounds. Subsequent fermentation may have further increased phenolic availability through the enzymatic degradation of cell wall components and the hydrolysis of bound phenolic complexes, resulting in the highest TPC values after 24 h fermentation [8].

#### 3.2.2. DPPH Radical Scavenging Activity

The DPPH radical scavenging activity ranged from 253.56 to 274.23 µmol TE/L and did not differ significantly among treatments or fermentation times (*p* > 0.05) (Table 2). Although slight fluctuations were observed, all samples remained within a relatively narrow range throughout the fermentation process. The highest DPPH activity was recorded in the control at 0 h (274.23 µmol TE/L), while the lowest value was observed in the 1.5 kV/cm treatment at 24 h (253.56 µmol TE/L). The lack of significant differences suggests that neither PEF treatment nor fermentation substantially altered the beverage’s free radical-scavenging capacity, as measured by the DPPH assay. This may indicate that although phenolic concentrations increased during fermentation, the specific phenolic compounds released or transformed did not contribute proportionally to DPPH radical neutralisation. It is also possible that degradation of certain antioxidant compounds occurred simultaneously with the release of new phenolic constituents, resulting in an overall stable antioxidant capacity. The lack of significant changes in DPPH radical scavenging activity despite increases in TPC and FRAP agrees with observations in fermented tef products, where fermentation enhanced TPC and FRAP but had limited effects on DPPH radical scavenging activity [29]. These findings suggest that fermentation may preferentially enhance antioxidant compounds with electron-donating capacity rather than radical-scavenging properties.

#### 3.2.3. Ferric Reducing Antioxidant Power (FRAP)

The FRAP values, as shown in Table 2, ranged from 362.49 to 467.55 µmol TE/L and increased significantly during fermentation (*p* < 0.05). The control sample at 0 h exhibited the lowest reducing power (362.49 µmol TE/L), while PEF-treated samples at the same time already showed significantly higher FRAP values (408.73–410.69 µmol TE/L). This indicates that PEF treatment enhanced the reducing antioxidant capacity immediately after processing. After 14 h of fermentation, FRAP values increased further, reaching 434.33–451.01 µmol TE/L. The highest FRAP value was observed in the 3.0 kV/cm treatment after 24 h (467.55 µmol TE/L), significantly higher than those of most other treatments. The increase in FRAP is consistent with the observed increase in total phenolic content, as phenolic compounds are important contributors to electron-donating and reducing activities. The enhanced FRAP values in PEF-treated samples suggest that electroporation promoted the release of antioxidant compounds, while fermentation further increased the availability of reducing substances through microbial biotransformation. The significant increase in FRAP values following PEF treatment and fermentation is consistent with findings reported by Ntourtoglou et al. [28] and Gao et al. [8] for rosehips and wheat-based beverages, where enhanced extraction of phenolic compounds resulted in improved antioxidant capacity, respectively. Similarly, PEF-assisted fermentation improved antioxidant capacity through the enhanced release of phenolic compounds and the generation of antioxidant metabolites in wheat-based beverages [8]. Although specific energies applied in the latter study were three times higher at 102 kJ/kg compared to our highest at 30 kJ/kg, the findings were similar. The strong relationship between TPC and FRAP suggests that the released phenolics substantially contributed to the beverage’s ferric-reducing power. The antioxidant results indicate that fermentation and PEF treatment positively influenced the beverage’s bioactive properties.

Total phenolic content increased significantly throughout fermentation, with the greatest enhancement observed in the 3.0 kV/cm PEF-treated sample after 24 h. Similarly, FRAP values increased significantly, demonstrating improved reducing antioxidant capacity and supporting the TPC findings. In contrast, DPPH radical scavenging activity remained relatively unchanged despite increases in phenolic content, suggesting that the antioxidant compounds generated during fermentation contributed more strongly to reducing power than to free radical scavenging activity [25]. Overall, the combination of PEF treatment and fermentation, particularly at 3.0 kV/cm for 24 h, appears to be the most effective strategy for enhancing the beverage’s phenolic composition and antioxidant potential. The increases in total phenolic content and ferric reducing antioxidant power observed in PEF-treated fermented beverages are consistent with findings reported for PEF-treated rosehips [28]. Pulsed electric field processing induces electroporation of plant tissues, enhancing membrane permeability and facilitating the release of intracellular bioactive compounds. During fermentation, microbial enzymatic activity may further hydrolyse cell-wall structures and liberate bound phenolics, leading to a progressive increase in TPC. The higher FRAP values observed in the 3.0 kV/cm treatment likely reflect the greater availability of reducing phenolic compounds released through the combined effects of PEF and fermentation.

In contrast, DPPH activity remained unchanged, suggesting that the phenolic compounds generated contributed more strongly to ferric ion reduction than to radical scavenging activity. These findings indicate that PEF pre-treatment can improve both the extractability of phenolic compounds and the functional properties of fermented beverages. Panagiotis et al. [3], who evaluated the effect of PEF treatment on yoghurt fermentation, stated that low-to-moderate intensity (1–3.67 kV/cm) treatment might induce reversible electroporation in the cell membrane, which accelerates microbial activity or bioactive compound extraction, obtaining fermented beverages in a shorter processing time with improved quality.

Beyond the laboratory data, the increase in TPC and FRAP highlights PEF’s real-world potential for enhancing beverage functionality. Because phenolic compounds play a direct role in reducing oxidative stress, boosting their extraction allows us to create higher-value products from low-cost agro-industrial side streams. For the food industry, this strategy turns rosehip seed meal into a functional beverage base, reducing waste while improving nutritional value. Although in vivo health effects and bioavailability remain to be tested, these initial antioxidant gains justify moving forward with sensory panels, shelf-life assessments, and clinical validation.

## 4. Study Limitations and Future Perspective

The study was conducted using a benchtop pulsed electric field (PEF) system, which limited the electric field strength we could test. With reference to filtration, the study relied on a practical laboratory method applying manual pressing using muslin cloths rather than an automated industrial setup. Although we kept this process constant across all runs, minor batch-to-batch variations were likely unavoidable. Additionally, we did not include a specific step to clear yeast cells post-fermentation, meaning residual yeast in the filtrate might have influenced the final physicochemical properties.

A few analytical limits also apply. We measured total phenolic content and antioxidant activity to gauge functional potential, but we did not run high-performance liquid chromatography (HPLC) to identify or quantify individual polyphenols. Nor did we conduct a formal sensory analysis. Detailed HPLC profiling would give a clearer picture of how PEF reshapes specific phytochemicals, while sensory testing is crucial to figure out consumer perception of the beverage.

To address these limitations, future research should focus on optimising the PEF processing conditions to establish an optimal treatment parameter that can be translated to a pilot-scale beverage production, adopting standardised filtration alongside controlled yeast removal. We also plan to map individual phenolic profiles using HPLC, evaluate shelf-life, and conduct formal sensory trials to gauge consumer appeal. Finally, assessing the bioavailability of these bioactive compounds will be key to understanding their actual health impact, providing the scientific backing needed to bring this sustainable functional beverage to market.

## 5. Conclusions

The results demonstrate that fermentation time had a more pronounced effect on the beverage’s physicochemical properties than PEF treatment did. Fermentation significantly reduced soluble solids and increased alcohol production, confirming active microbial metabolism. In contrast, pH remained relatively stable throughout the process. Colour measurements revealed that fermentation progressively increased lightness while reducing both red and yellow colour intensities. Although some numerical differences were observed between the control and PEF-treated samples, the effects of PEF on physicochemical properties were generally limited compared with the influence of fermentation.

PEF treatment yielded significant improvements in both TPC and FRAP values, whereas DPPH radical scavenging activity showed no statistically significant differences across treatments. Such discrepancies between assays are well-documented in PEF-processed plant tissues. Because FRAP measures electron transfer to ferric ions while DPPH relies on radical quenching kinetics, structural shifts in specific phenolics can boost ferric reducing power without substantially altering DPPH values. Thus, the primary evidence for the beverage’s improved functional potential lies in its increased phenolic content and enhanced FRAP activity.

The findings demonstrate that PEF treatment can be successfully applied to valorise rosehip seed meal with added baobab to produce a fermented beverage with enhanced antioxidant properties. This approach offers a sustainable strategy for converting an underutilised agro-industrial waste into a beverage, with potential applications in the functional food industry and the circular bioeconomy.

## Figures and Tables

**Table 1:** Physicochemical properties of PEF-treated rosehip seed meal and baobab pulp fermented beverage.

Sample	pH	°Brix	%ABV	L*	a*	b*
RSM_C_T0_	3.75 ± 0.11 ^a^	10.40 ± 0.00 ^b^	0.03 ± 0.06 ^a^	36.29 ± 1.45 ^b^	3.57 ± 1.4 ^cd^	20.80 ± 1.92 ^d^
RSM_1.5_T0_	3.75 ± 0.10 ^a^	10.40 ± 0.00 ^b^	0.04 ± 0.07 ^a^	36.55 ± 0.37 ^b^	2.94 ± 0.12 ^bcd^	16.92 ± 1.17 ^bc^
RSM_3.0_T0_	3.76 ± 0.10 ^a^	10.53 ± 0.12 ^b^	0.04 ± 0.08 ^a^	34.70 ± 0.82 ^a^	3.65 ± 1.36 ^d^	17.87 ± 1.95 ^c^
RSM_C_T14_	3.71 ± 0.02 ^a^	9.79 ± 1.82 ^b^	0.82 ± 0.03 ^b^	36.89 ± 0.20 ^bc^	2.29 ± 0.49 ^bcd^	13.74 ± 1.32 ^a^
RSM_1.5_T14_	3.73 ± 0.01 ^a^	9.92 ± 0.09 ^b^	0.80 ± 0.01 ^b^	38.11 ± 0.23 ^c^	2.84 ± 0.83 ^bcd^	14.45 ± 0.80 ^ab^
RSM_3.0_T14_	3.68 ± 0.08 ^a^	9.77 ± 0.23 ^b^	0.85 ± 0.06 ^b^	38.17 ± 0.42 ^c^	1.59 ± 0.07 ^ab^	13.96 ± 0.69 ^a^
RSM_C_T24_	3.67 ± 0.06 ^a^	8.33 ± 0.61 ^a^	1.28 ± 0.21 ^c^	43.14 ± 1.36 ^e^	1.47 ± 0.62 ^ab^	15.87 ± 1.08 ^abc^
RSM_1.5_T24_	3.69 ± 0.04 ^a^	8.23 ± 0.75 ^a^	1.42 ± 0.03 ^c^	43.68 ± 0.69 ^e^	2.10 ± 0.50 ^abc^	15.22 ± 2.31 ^abc^
RSM_3.0_T24_	3.67 ± 0.08 ^a^	8.43 ± 0.68 ^a^	1.35 ± 0.08 ^c^	41.33 ± 0.97 ^d^	0.76 ± 0.21 ^a^	13.65 ± 0.69 ^a^

Data presented as mean ± standard deviation (*n* = 3) of pH, total soluble solids (°Brix), alcohol by volume (%ABV), lightness (L*), red/green (a*), yellow/blue (b*) of rosehip seed meal and baobab pulp fermented beverage. Rosehip seed meal (RSM); Control 0 h fermentation (C_T0_); PEF treatment 1.5 kv/cm 0 h fermentation (1.5_T0_); PEF treatment 3 kv/cm 0 h fermentation (3.0_T0_); Control 14 h fermentation (C_T14_); PEF treatment 1.5 kv/cm 14 h fermentation (1.5_T14_); PEF treatment 3 kv/cm 14 h fermentation (3.0_T14_); Control 24 h fermentation (C_T24_); PEF treatment 1.5 kv/cm 24 h fermentation (1.5_T24_); PEF treatment 3 kv/cm 24 h fermentation (3.0_T24_). ^a–e^ Means with different letter superscripts in the same column denote significant differences (*p* < 0.05).

**Table 2:** Total phenolic content and antioxidant activity of PEF-treated rosehip seed meal and baobab pulp fermented beverage.

Sample	TPC	DPPH	FRAP
RSM_C_T0_	499.30 ± 23.63 ^a^	274.23 ± 35.00 ^a^	362.49 ± 3.21 ^a^
RSM_1.5_T0_	507.23 ± 25.79 ^a^	256.68 ± 25.42 ^a^	408.73 ± 11.37 ^b^
RSM_3.0_T0_	520.00 ± 7.00 ^ab^	261.00 ±18.25 ^a^	410.69 ± 8.33 ^bc^
RSM_C_T14_	553.00 ± 27.62 ^bc^	262.70 ± 20.21 ^a^	442.13 ± 33.98 ^cde^
RSM_1.5_T14_	564.00 ± 24.30 ^cd^	267.59 ± 27.01 ^a^	434.33 ± 8.96 ^bcd^
RSM_3.0_T14_	570.67 ± 23.10 ^cd^	273.66 ± 25.97 ^a^	451.01 ± 24.56 ^de^
RSM_C_T24_	578.33 ± 14.19 ^cd^	268.00 ±19.00 ^a^	428.29 ± 13.58 ^bcd^
RSM_1.5_T24_	554.01 ± 18.33 ^bc^	253.56 ± 20.55 ^a^	414.37 ± 8.02 ^bc^
RSM_3.0_T24_	602.41 ± 19.73 ^d^	268.33 ± 21.73 ^a^	467.55 ± 18.15 ^e^

Data presented as mean ± standard deviation (*n* = 3) of phenolic content and antioxidant activity of rosehip seed meal and baobab pulp fermented beverage. Rosehip seed meal (RSM); Control 0 h fermentation (C_T0_); PEF treatment 1.5 kv/cm 0 h fermentation (1.5_T0_); PEF treatment 3 kv/cm 0 h fermentation (3.0_T0_); Control 14 h fermentation (C_T14_); PEF treatment 1.5 kv/cm 14 h fermentation (1.5_T14_); PEF treatment 3 kv/cm 14 h fermentation (3.0_T14_); Control 24 h fermentation (C_T24_); PEF treatment 1.5 kv/cm 24 h fermentation (1.5_T24_); PEF treatment 3 kv/cm 24 h fermentation (3.0_T24_). ^a–e^ Means with different letter superscripts in the same column denote significant differences (*p* < 0.05).

## Data Availability

The data presented in this study are available on request from the corresponding author.
